# Effect of Corrosive Aging Environments on the Flexural Properties of Silane-Coupling-Agent-Modified Basalt-Fiber-Reinforced Composites

**DOI:** 10.3390/ma16041543

**Published:** 2023-02-12

**Authors:** Xuanyao Luo, Yuehai Wei, Leilei Ma, Wei Tian, Chengyan Zhu

**Affiliations:** 1 Key Laboratory of Advanced Textile Materials and Preparation Technology of the Ministry of Education, College of Textiles Science and Engineering, Zhejiang Sci-Tech University, Xiasha Campus, Hangzhou 310018, China; 2 Zhejiang Sci-Tech University Huzhou Research Institute Co., Ltd., Huzhou 313000, China

**Keywords:** BFRP, silane coupling agent, environmental degradation, interface property, flexural properties

## Abstract

In recent years, basalt-fiber-reinforced polymers (BFRPs) have been widely used in the field of corrosive aging resistance. In this paper, BFRPs are made into composite laminates, and the flexural properties of BFRPs modified with different types of silane coupling agents, KH550 (aminopropyl-triethoxysilane), KH560 (glycidyletheroxypropyl-trimethoxysilane), and A171 (vinyl-trimethoxysilane), immersed at 20 °C, 40 °C, and 60 °C in a 3.5% NaCl concentration artificial seawater, a 10% NaCl high-concentration artificial seawater, 10% H_2_SO_4_, or 10% NaOH are investigated. The results show that the flexural strength decreased with increasing exposure time in corrosive aging environments at different temperatures. The temperature greatly influences flexural strength, and the flexural strength decreases rapidly in high-temperature acidic and alkaline environments. In addition, we found that the flexural retention in the seawater environment did not change much compared to that in the water environment, indicating that BFRPs have relatively good resistance to seawater corrosion. The silane coupling agent modification enhances flexural strength and flexural strength retention by enhancing the interfacial bonding property of the BFRPs. Considering the experimental results, the three silane coupling agents modified the corrosive aging performance of the composites in the order of KH550 > KH560 > A171. This will provide theoretical support for the application of silane-coupling-agent-modified BFRPs in corrosive aging environments.

## 1. Introduction

Due to the advantages of high specific strength and modulus and low weight compared with traditional metal and non-metal materials, fiber-reinforced polymers (FRPs) are widely used in automotive, railroad, aerospace, wind energy, and marine fields [1,2,3,4,5,6,7,8]. However, FRPs face challenges in different environments such as moisture, high temperature, seawater, acidic and alkaline, UV, and other corrosive aging environments [9,10]. The physical, chemical, and mechanical properties of FRPs are often degraded after moisture absorption in a corrosive aging environment [11]. During the degradation of composite properties, the resin matrix undergoes plasticization, swelling, macromolecular chain breakage, and hydrolysis, resulting in internal stresses at the composite interface that deteriorates the interfacial bonding conditions [12,13]. In addition, chemical damage caused by hydrolysis reactions at the substrate and interface can weaken the interfacial adhesion properties [14,15]. The debonding of the interface provides a favorable site for the occurrence of capillary phenomena, which accelerate the penetration and diffusion of water molecules into the interior of the material and promote the expansion of microcracks in the interface and matrix, which likewise accelerates the degradation and performance degradation of the composite material [16,17].

Many scholars have conducted many studies on the property changes of FRPs under corrosive aging environments. Yan et al. [18] revealed the changes in the properties of flax-fiber-reinforced resin matrix composites after corrosive aging in alkali, seawater, and water environments. The results showed that all aging solutions resulted in the composites experiencing severe degradation and a significant decrease in the tensile/flexural properties of the flax fabric/epoxy composites. Bazlie et al. [19] investigated the changes in the mechanical properties of GFRP under corrosion in seawater, acidic, and alkaline environments. It was found that the mechanical strength of composites immersed in alkaline solutions decreased more compared to seawater and acidic solutions. Feng et al. [20] investigated the long-term performance changes of GFRP in highly corrosive environments. GFRP samples were exposed to different concentrations of alkaline, saline, and acidic solutions at 60 °C. In addition, the effect of acidic solutions at high temperatures (90 °C) was also investigated. The results showed that the flexural strength of acidic and alkaline solutions decreased with increasing exposure time. The flexural properties decreased significantly at high concentrations of acidity and high temperatures.

The durability and reliability of FRPs in corrosive aging environments are very important practical issues for this material. The structural integrity and lifetime performance of the composites strongly depend on the stability of the fiber–resin matrix interface region [21]. FRPs prepared from fibers treated with silane coupling agents have been reported to exhibit less degradation in performance than untreated composites after long-term exposure to corrosive environments [22]. The use of silane coupling agents improves the mechanical interlocking and chemical interactions between fibers and the resin matrix, effectively enhancing the interfacial bonding between fibers and the resin matrix. Wang et al. [23] reported that the water resistance and damping properties of the composite were improved by treating the flax-fiber-reinforced resin matrix composites with a silane coupling agent. Hill et al. [24] reported that acetylation and silylation can protect nonwoven or oil palm hollow-fruit-bundle-fiber-mat-reinforced polyester matrix composites from the deterioration of mechanical properties after exposure to corrosive environments. Tual et al. [25] observed that the mechanical properties of carbon/epoxy composites are reduced during accelerated seawater aging. Mittal et al. [26] found that the mechanical properties of seawater-aged composites were improved using 3-aminopropyltriethoxysiliane-treated MMT compared to untreated MMT-modified glass-fiber-reinforced vinyl ester resin composites.

The results of existing studies provide many insights into the properties of composites under corrosive aging environments, especially for glass-fiber and natural-fiber-reinforced composites, but relatively few studies have been conducted on the long-term performance changes of basalt-fiber-reinforced resin matrix composites under corrosive aging environments. Basalt fibers are one of the most widely used high-performance green inorganic fibers in various industries [27]. Due to its unique corrosion resistance, high fire performance, heat and sound insulation, environmental protection, high toughness, etc., it occupies an important position in civil, agricultural, defense industries, and aviation applications, and basalt fiber can be selected to replace glass fiber in many engineering applications to make composite materials [28,29]. Therefore, in this paper, the effect of corrosive aging time and immersion temperature in water, seawater, high-concentration seawater, acid, and alkali environments on the flexural properties of silane coupling agents modified BFRPs were investigated.

## 2. Materials and Methods

### 2.1. Materials

The sample used is basalt fiber plain fabric, with a warp and weft density of 50 pieces/10 cm, a surface density of 300 g/m^2^, and a surface presenting a similar black-gold color, produced by Haining Anjie Composites Co. (Jiaxing, China). Polyimide film was produced by Shenzhen Runhai Electronics Co. (Shenzhen, China). Vinyl ester resin was produced by Shangwei New Material Technology Co. (Shanghai, China). Acetone was produced by Xilong Science Co. (Shanghai, China). Silane coupling agents KH550, KH560, and A171, anhydrous ethanol, deionized water, sodium chloride, 10% dilute sulfuric acid, and 10% sodium hydroxide were produced by Hangzhou High Crystal Fine Chemical Co. (Hangzhou, China).

### 2.2. Preparation of BFRPs

**Figure 1:**
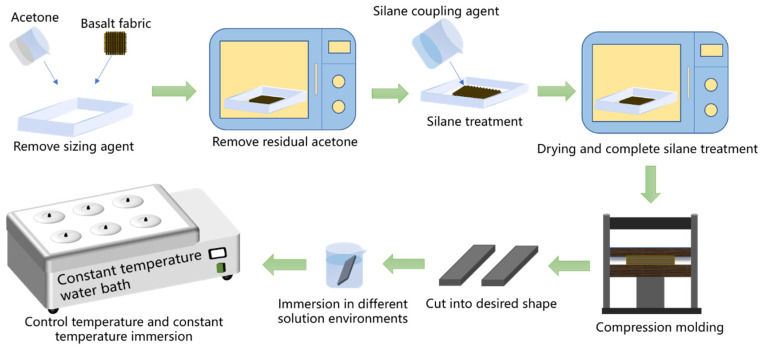
The preparation process of BFRPs.

**Figure 2:**
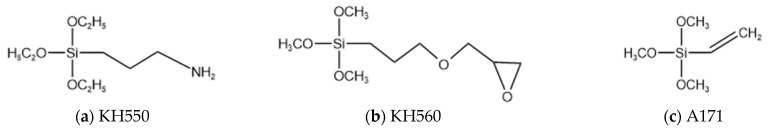
Chemical structures of different silane coupling agents.

The preparation process of BFRP samples is shown in Figure 1. Firstly, the basalt fibers were pretreated, and the basalt fiber plain fabrics were soaked in acetone solution for 48 h to remove the surface sizing agent and then washed with deionized water several times and put into a vacuum oven at 80 °C to dry them thoroughly [30]. Then, we used the silane coupling agents KH550, KH560, and A171 to modify the basalt fibers, with anhydrous ethanol and deionized water as a solvent in a ratio of 8:2, mixed to prepare a mass fraction of 1% of the alcohol solution to the silane coupling agent. The above treatments of the fabrics were placed in it at room temperature. After 5 h of reaction, anhydrous ethanol was used to wash away the unreacted silane coupling agent, and then the samples were put into a vacuum oven at 80 °C. The chemical structures of the silane coupling agents KH550, KH560, and A171 are shown in Figure 2 [31]. Finally, six layers of basalt fiber plain fabric were molded with vinyl ester resin using a semi-automatic flat vulcanizer. The curing conditions were set as follows: 30 °C, 60 min, 2 MPa pressure, 50% fiber mass fraction in the composite, and 2 mm sheet thickness.

### 2.3. Immersion Experiment

**Figure 3:**
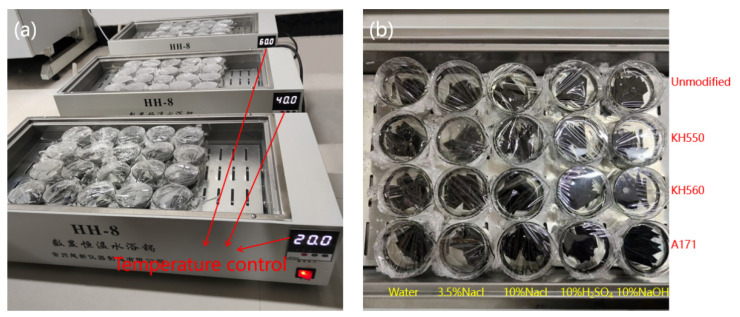
Immersion experiment: (**a**) immersion experiment equipment; (**b**) close-up view of immersed samples.

This study used deionized water, 3.5% NaCl concentration artificial seawater, 10% NaCl high-concentration artificial seawater, 10% H_2_SO_4_ acid, and 10% NaOH alkali as the five corrosive aging environments selected (refer to Table 1). First, the samples were completely immersed in the test conditions. When the temperature reached the required level for each test condition, the samples were inserted, and the start of the immersion period was recorded. In addition, these conditions needed to remain constant throughout the immersion period. Samples were removed from each solution and tested, and the diagram of the immersion experiment is shown in Figure 3.

### 2.4. SEM Imaging

The section morphology of BFRPs modified with different types of silane coupling agents was observed by GeminiSEM-500 field emission scanning electron microscopy with a magnification of 3000.

### 2.5. Mass Change

Before the immersion of the samples in corrosive aging environments, all BFRP samples were dried in an oven at 60 °C for 48 h until the weight no longer changed. The mass change was detected by periodically weighing the samples in the immersion bath. Residual water trapped on the surface of the samples was wiped with a paper towel before weighing. After that, the weight of the samples was recorded at different time intervals during aging using an electronic balance with an accuracy of 0.1 mg. Five samples were measured for each condition. The mass change of the BFRPs test was defined as:

$$M_{t}(\%)=\frac{W_{t-}W_{0}}{W_{0}}\times 100$$

where $W_{0}$ is the initial weight of the sample and $W_{0}$ is the weight of the sample after immersion.

### 2.6. Three-Point Flexural Test

**Figure 4:**
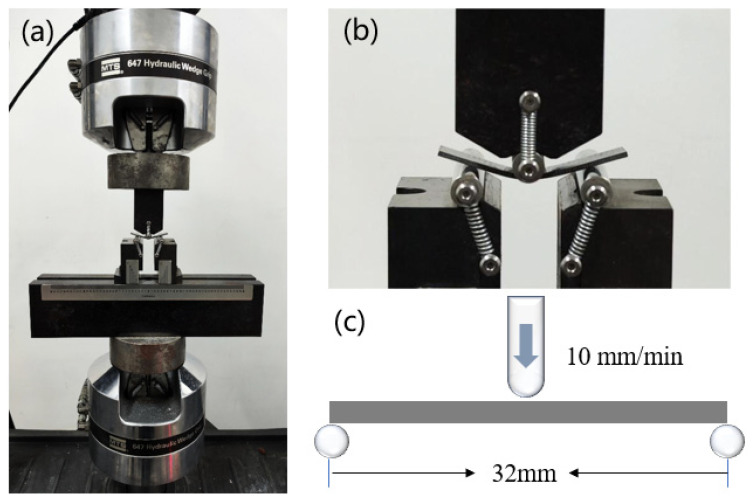
Three-point flexural test diagrams: (**a**) sample under flexural load; (**b**) sample fracture closer view; (**c**) schematic diagram.

Samples of 60 × 15 × 2 mm were cut out from the middle of each group of BFRPs and a total of five samples were obtained. The samples were placed on a universal testing machine (M.T.S. landmark 370.10, Eden Prairie, MN, USA) to test the three-point flexural performance (shown in Figure 4). The span was set to 32 mm, and the upper indenter remained stationary. The lower indenter is loaded upwards at a speed of 5 mm/min so that the samples were stressed at three points, and finally, a fracture occurred in the middle of the sample. The test was carried out under the conditions of a temperature of 25 °C and a humidity of 50%. The flexural strength was calculated as follows:

$$\delta_{f}=\frac{3P\times l}{\;2b\times h^{2}}$$

where $\delta_{f}$ is the flexural strength in MPa (MPa); P is the failure load in Newtons (N); l is the span in mm; h is the thickness of the sample in mm; and b is the width of the sample in mm, taking the average of five samples. The flexural strength retention rate was calculated as:

$$R\left(\%\right)=\frac{H}{H_{0}}$$

where H is the flexural strength of the BFRPs after immersion, $H_{0}$ is the flexural strength of the BFRP before immersion in MPa, and five samples are taken for the average value.

## 3. Results and Discussion

### 3.1. SEM Imaging

**Figure 5:**
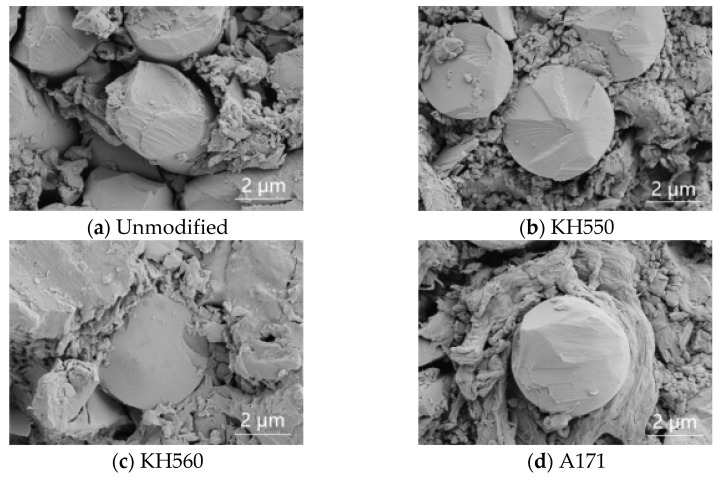
Cross-sectional view of BFRPs modified with different types of silane coupling agents.

**Figure 6:**
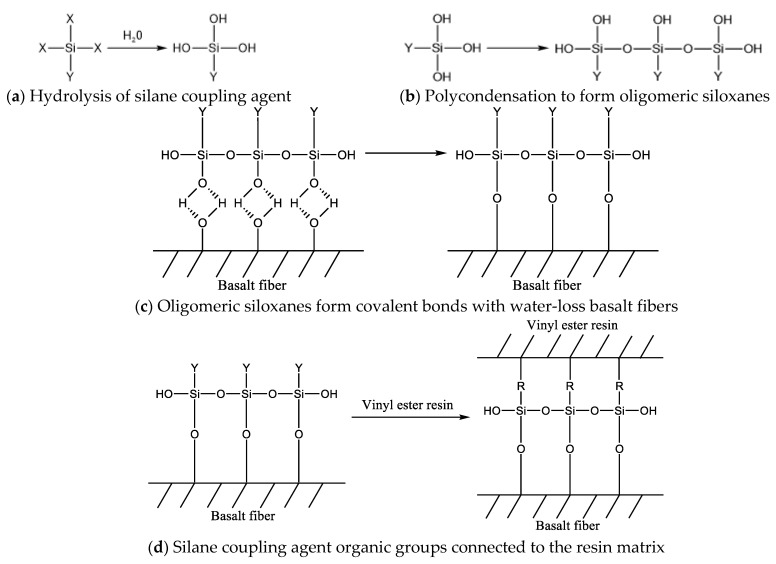
Mechanism of action of a silane-coupling-agent-modified BFRP.

As can be seen from Figure 5, the interfacial bonding effect of the BFRPs was improved after the modification treatment with silane coupling agents KH550, KH560, and A171. When the BFRPs were not modified with silane coupling agents, there was a large gap between the fibers and the resin matrix, and the interfacial bonding effect was poor. A silane coupling agent is a compound that connects hydrolysis group X and organic group Y with different chemical properties to the same silicon atom. After modification with silane coupling agents KH550, KH560, and A171, the silane coupling agent hydrolysis of the formation of siloxane will be grafted and adsorbed on the surface of basalt fibers and form covalent bonds, and the organic polymer molecules of vinyl ester resin will react with the organic groups of siloxanes to make fiber–resin matrix bonds. Meanwhile, the silane coupling agent modification enhances the interfacial adhesion between fiber and resin and makes the basalt fiber and vinyl ester resin matrix more closely bonded to each other. The mechanism of action is shown in Figure 6 [31].

### 3.2. Change in Appearance

**Figure 7:**
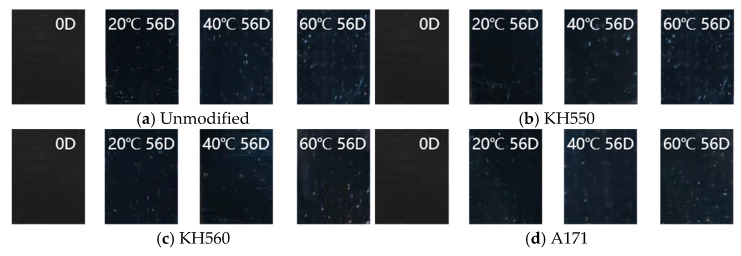
Appearance changes in a water environment.

**Figure 8:**
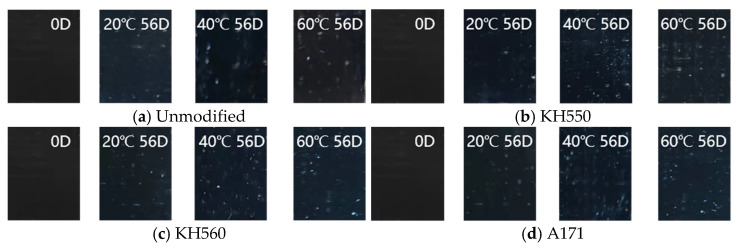
Appearance changes in a seawater environment.

**Figure 9:**
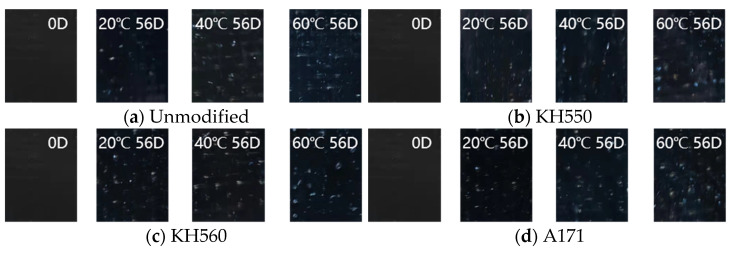
Appearance changes in a high-concentration seawater environment.

**Figure 10:**
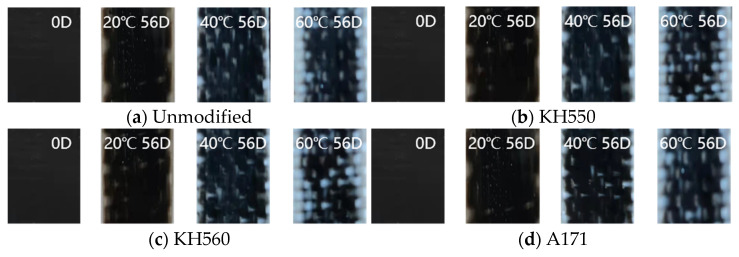
Appearance changes in a 10% H_2_SO_4_ acid environment.

**Figure 11:**
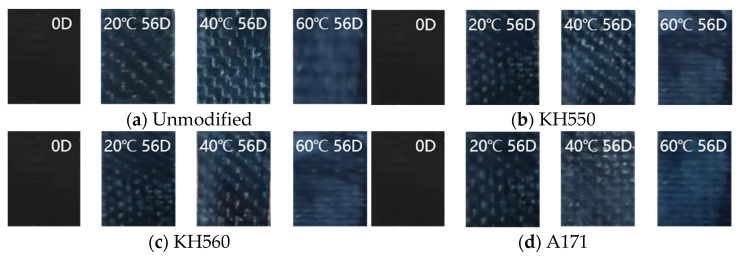
Appearance changes in a 10% NaOH alkali environment.

As shown in Figure 7, Figure 8, Figure 9, Figure 10 and Figure 11 for the samples immersed in different temperatures and different environments after 56 days of appearance change, the appearance of the samples immersed in 20 °C environments was almost unchanged; for samples immersed in 50 °C acidic and alkaline environments, the color of the sample began to change; and in 80 °C acidic and alkaline environments, with the dissolution of the surface resin matrix, the color changed more obviously. Soaked in different temperatures of water and seawater environments, the sample appearance changes were very small, indicating that the samples had good resistance to heat, moisture, and seawater’s corrosive aging properties. The appearance of the samples immersed in acidic and alkaline environments changed most obviously, and a large number of white patches appeared on the samples. Different silane coupling agent modifications did not have much effect on the sample appearances. This paper will further explore the effect of different environments and temperatures on the flexural properties of silane-coupling-agent-modified samples and explore the corrosion and aging mechanism.

### 3.3. MASS Changes in Different Immersion Environments

**Figure 12:**
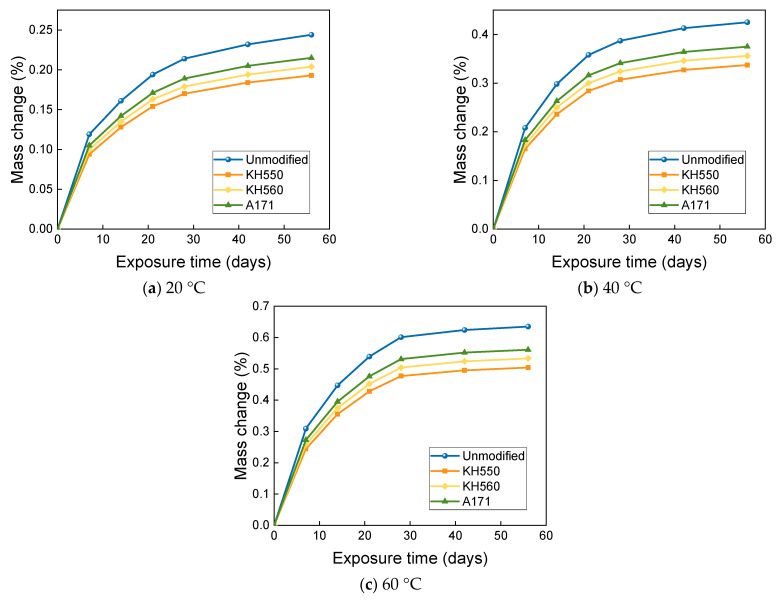
Mass change in a water environment.

From Figure 12, it can be seen that the mass change of BFRPs immersed in water environments at different temperatures follows a two-stage rule. The mass of the BFRPs grew rapidly in the initial stage of immersion, and the mass of the BFRPs still grew in the subsequent stage, but the growth rate kept decreasing. After 56 days of immersion in water environments at 20 °C, the mass change of unmodified and KH550, KH560, and A171 silane-coupling-agent-modified BFRPs was 0.244%, 0.193%, 0.204%, and 0.215%; after 56 days of immersion in water environments at 40 °C, the mass change of unmodified and KH550, KH560, and A171 silane-coupling-agent-modified BFRPs was 0.425%, 0.337%, 0.356%, and 0.375%. For the unmodified and KH550, KH560, and A171 silane-coupling-agent-modified BFRPs after 56 days of immersion in water environments at 60 °C, the mass change was 0.635%, 0.504%, 0.533%, and 0.561%. In general, the mass growth or loss of the BFRPs under different corrosive aging environments accumulates and the rate of change gradually decreases with the increase in time. The mass change of BFRPs is mainly affected by the water absorption and hydrolysis of vinyl ester resin, which is hydrolyzed and decomposed into a low-molecular-weight polymer [32]. When the mass increase due to water absorption exceeds the mass loss due to the hydrolytic decomposition of the resin matrix, an overall mass increase is observed. The mass change of BFRPs modified by silane coupling agents KH550, KH560, and A171 is low compared to the unmodified BFRP. This is because, when modified with a silane coupling agent, the hydroxyl group of the hydrophilic group on the fiber surface reacts with siloxane and reduces its hydrophilicity, and the organic polymer molecules of vinyl ester also react with the organic group of siloxanes and connect, and the fiber–resin matrix bonding can reduce the water absorption of BFRPs [33]. In addition, the silane coupling agent modification helps to improve the adhesion between the fibers and the matrix, and the gap in the interfacial region is reduced, resulting in lower water absorption. When the temperature is increased, the mass growth rate of BFRPs in water environments increases significantly, which may be due to the more intense movement of water molecules at higher temperatures and the higher void pressure due to the increase in gas volume within BFRPs [34]. The increase in pressure favors the extension of microcracks, thus increasing the free volume inside the sample, which can be filled by the surrounding solution, thus allowing the resin matrix to absorb more water [35].

**Figure 13:**
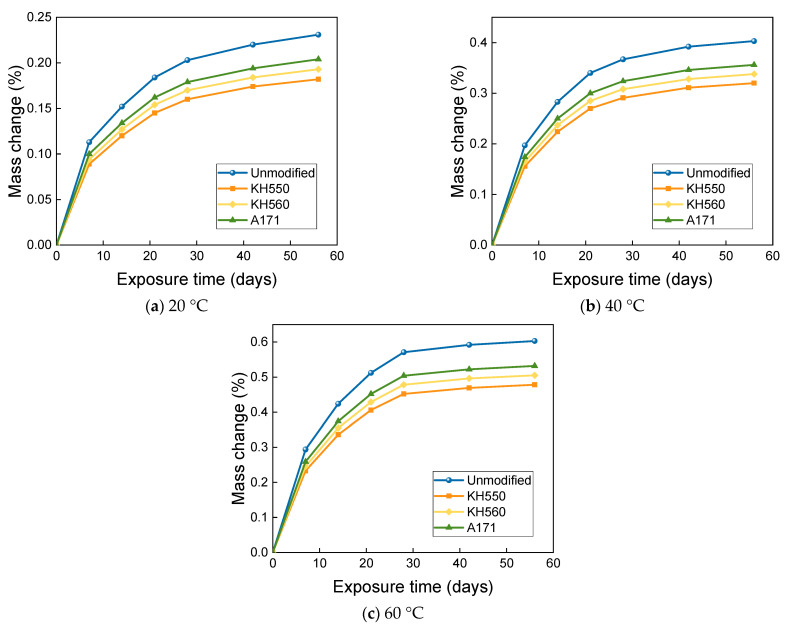
Mass change in a 3.5% NaCl seawater environment.

**Figure 14:**
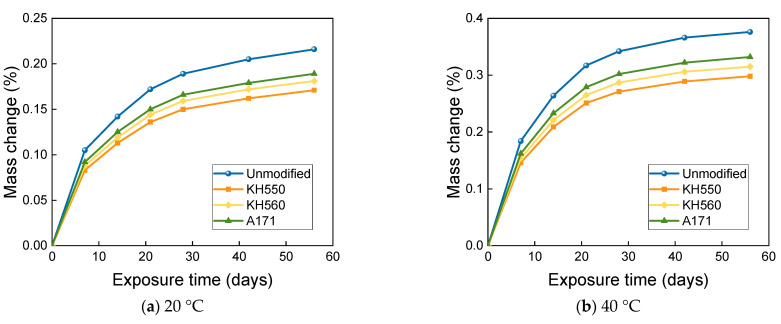

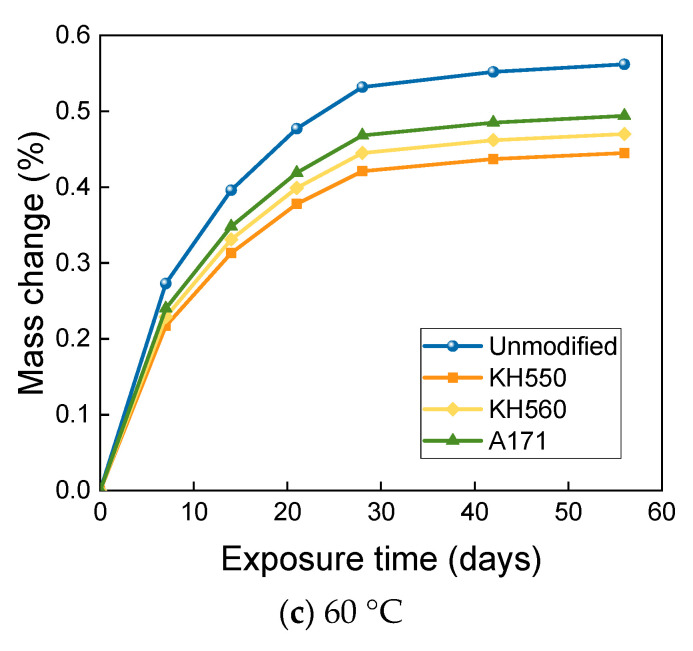
Mass change in a 10% NaCl high-concentration seawater environment.

The mass changes of BFRPs under different temperatures in 3.5% NaCl seawater environments are shown in Figure 13. The mass growth was rapid at the early stage of immersion, and the mass growth rate leveled off in the middle of immersion but showed an overall increasing trend. After soaking for 56 days in 20 °C seawater environments, the mass of unmodified and KH550, KH560, and A171 silane-coupling-agent-modified BFRPs increased by 0.231%, 0.182%, 0.193%, and 0.204%; after soaking for 56 days in 40 °C seawater environments, the mass of unmodified and KH550, KH560, and A171 silane-coupling-agent-modified BFRPs increased by 0.403%, 0.320%, 0.338%, and 0.356%. The mass of BFRPs modified with KH550, KH560, and A171 silane coupling agents increased by 0.603%, 0.478%, 0.505%, and 0.532% after 56 days of immersion in seawater environments at 60 °C. As shown in Figure 14, the mass change of BFRPs under immersion in 10% NaCl high-concentration seawater environments at different temperatures followed a two-stage pattern. The mass of the BFRPs grew rapidly in the initial stage of immersion, and the mass of BFRPs still grew in the subsequent stages, but the growth rate kept decreasing. After 56 days of immersion in 20 °C seawater environments, the mass of unmodified and KH550, KH560, and A171 silane-coupling-agent-modified BFRPs increased by 0.216%, 0.171%, 0.181%, and 0.189%; after 56 days of immersion in a seawater environment at 40 °C, the masses of the unmodified and KH550, KH560, and A171 silane-coupling-agent-modified composites increased by 0.376%, 0.298%, 0.315%, and 0.332%; after 56 days of immersion in a seawater environment at 60 °C, the masses of the unmodified and KH550, KH560, and A171 silane-coupling-agent-modified composites increased by 0.562%, 0.445%, 0.470%, and 0.494%.

The mass growth rate of BFRPs after immersion in seawater is less than that after water immersion. This is because the presence of salt in seawater reduces the activity of water molecules since salt particles appearing in seawater are less absorbable than water. This leads to the accumulation of salt particles on the sample surface, making the concentration of salt particles in seawater inside the sample smaller than that in seawater, which creates an osmotic pressure that further inhibits water absorption [36].

**Figure 15:**
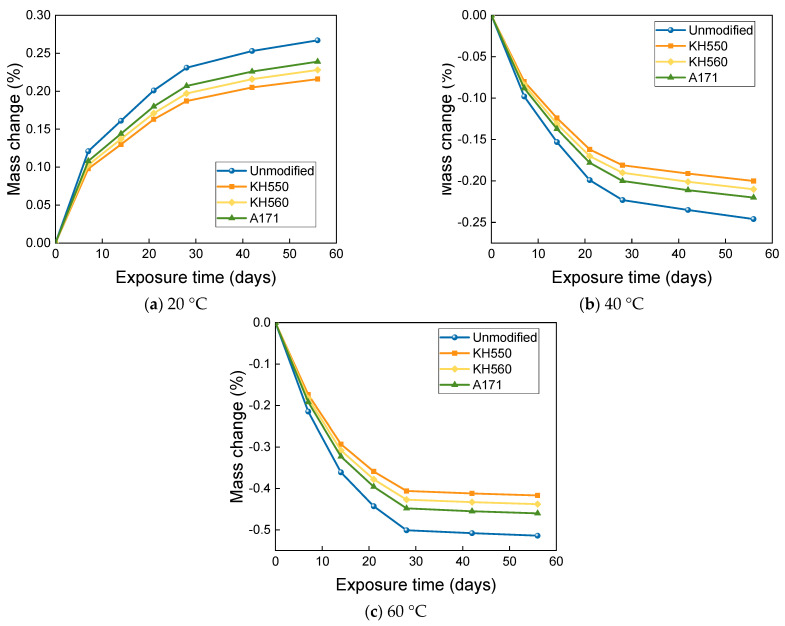
Mass change in a 10% H_2_SO_4_ acidic environment.

The mass change of BFRPs after soaking in 10% H_2_SO_4_ acidic environments at different temperatures is shown in Figure 15. When the immersion temperature was 20 °C, the mass of BFRPs increased rapidly in the first period with the soaking time, while the growth was relatively slow in the later period. After 56 days of immersion in acidic environments at 20 °C, the mass of unmodified and KH550, KH560, and A171 silane-coupling-agent-modified BFRPs increased by 0.267%, 0.216%, 0.228%, and 0.239%; when the immersion temperature was further increased to 40 °C and 60 °C, the mass of BFRPs decreased rapidly with the increase in the immersion time in the first period, while the later decrease was relatively slow. The mass of unmodified and KH550, KH560, and A171 silane-coupling-agent-modified BFRPs decreased by 0.246%, 0.200%, 0.210%, and 0.220% after 56 days of immersion in 40 °C seawater environments; after 56 days of immersion in 60 °C seawater environments, the mass of unmodified and KH550, KH560, and A171 silane-coupling-agent-modified BFRPs decreased by 0.514%, 0.417%, 0.438%, and 0.460%. It could be found that the mass of BFRPs showed a decrease when the temperature was increased to 40 °C and 60 °C. This is due to the intense movement of water molecules after the temperature increase, and the overall mass decrease in BFRPs is observed when the mass increase due to water absorption is smaller than the mass loss due to the leaching of low-molecular-weight polymers [37].

**Figure 16:**
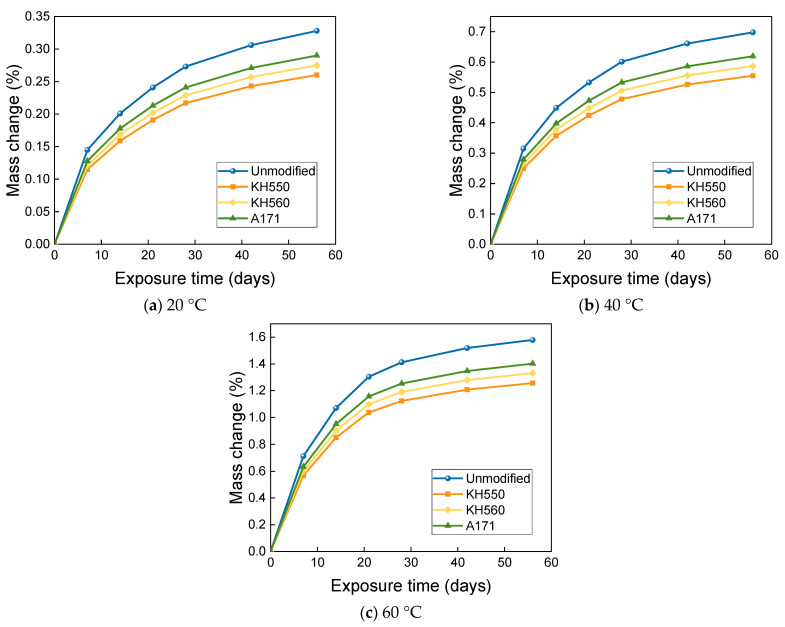
Mass change in a 10% NaOH alkaline environment.

As can be seen from Figure 16, the mass change of BFRPs soaked in 10% NaOH alkaline environments at different temperatures increased very rapidly at the initial stage of soaking, and the mass of the BFRPs still increased at the later stage of soaking, but the growth rate slowed down. After 56 days of immersion in alkaline environments at 20 °C, the mass of BFRPs unmodified and modified by KH550, KH560, and A171 silane coupling agents increased by 0.328%, 0.260%, 0.275%, and 0.290%; after 56 days of immersion in alkaline environments at 40 °C, the mass of unmodified and KH550, KH560, and A171 silane-coupling-agent-modified BFRPs increased by 0.698%, 0.555%, 0.587%, and 0.619%; the masses of BFRPs unmodified and modified by KH550, KH560, and A171 silane coupling agents increased by 1.579%, 1.256%, 1.331%, and 1.402% after 56 days of immersion in alkaline environments at 60 °C. The mass change of BFRPs immersed in the alkaline environments was greater than in all other corrosive aging environments. This may be due to the penetration of the high pH solution, resulting in more microcracks and greater moisture uptake, which leads to greater weight gain. Similar results were reported by Karbhari and Chu [38], who compared the weight gain of glass/vinyl ester composites submerged in different solutions. When the composite samples were submerged in water, seawater, and alkaline solutions, the water molecules in these solutions penetrated the composites, increasing the mass of the samples.

### 3.4. Flexural Properties in Different Immersion Environments

**Figure 17:**
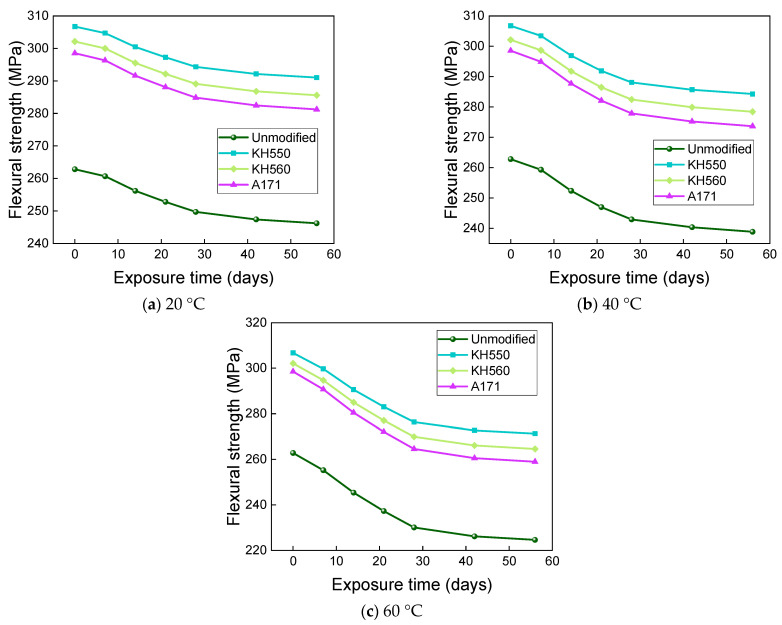
Flexural strength in a water environment.

**Figure 18:**
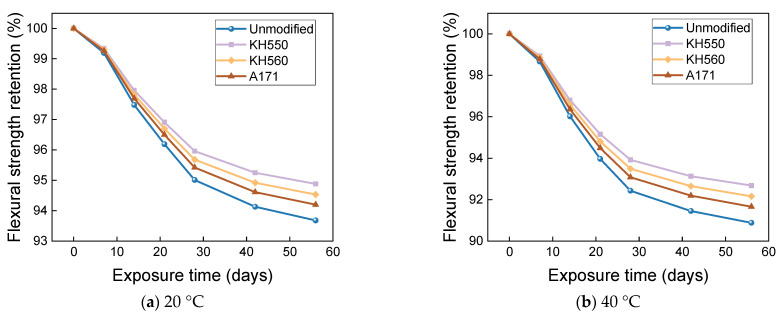

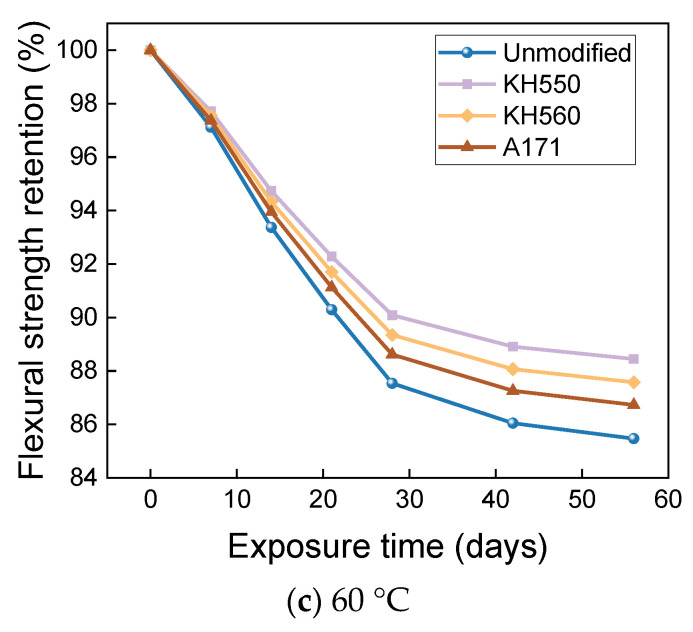
Flexural strength retention in a water environment.

The effects of water environments at different temperatures on the flexural strength and flexural strength retention of the samples are shown in Figure 17 and Figure 18. The flexural strength and retention of samples immersed in water environments at different temperatures showed a decreasing trend with time. After 56 days of immersion in 20 °C water environments, the flexural strengths of unmodified and KH550, KH560, and A171 silane-coupling-agent-modified BFRPs were 246.20 MPa, 291.03 MPa, 285.60 MPa, and 281.21 MPa, which decreased by 6.32%, 5.12%, 5.47%, and 5.80% compared with the flexural strength before immersion. The flexural strengths of the unmodified BFRPs and those modified with KH550, KH560, and A171 silane coupling agents were 238.84 MPa, 284.27 MPa, 278.42 MPa, and 273.65 MPa after 56 days of immersion in water at 40 °C, which were 9.12%, 7.32%, 7.84%, and 8.34% lower than the flexural strengths before immersion; after 56 days of immersion in water at 60 °C, the flexural strengths of the unmodified and KH550, KH560, and A171 silane-coupling-agent-modified BFRPs were 224.62 MPa, 271.30 MPa, 264.59 MPa, and 258.93 MPa, which decreased by 14.53%, 11.55%, 12.42%, and 13.27%. The entry of water into the resin matrix at the beginning of the immersion leads to the release of residual stresses in the matrix and the acceleration of post-curing, both of which slow down the rate of decline in the flexural strength of the BFRPs [39]. With the growth of soaking time, water molecules penetrate further and diffuse into the interior of the BFRPs, but the difference in the degree of moisture absorption and expansion of the matrix and fibers will lead to shear stress at the interface. When the shear stress exceeds the interface bonding force, cracks will occur, resulting in interface damage, which will reduce the efficiency of stress transfer between the resin and fibers, resulting in a decrease in the flexural strength of the samples [40,41]. When water comes into contact with the matrix, it undergoes a hydrolysis reaction. This decreases the density of the matrix, increases the number of pores, and reduces the adhesion to the fibers, which can affect the flexural properties of the BFRPs [42].

When the temperature increases, the flexural strength of the BFRPs decreases significantly, because the increase in temperature accelerates the hydrolysis reaction, generates higher void pressure, facilitates the expansion of microcracks in the BFRPs, and promotes more water entry. However, the increase in temperature also promotes the plasticization of the matrix by water. The long molecular chains of the resin matrix are hydrolyzed and decomposed and the bonding at the interface is weakened, which reduces the flexural strength of the BFRPs [43,44]. In addition, the flexural strength does not decrease linearly with time, and the rate of loss of flexural strength tends to decrease with increasing time. This may be due to the rapid hydrolysis reaction of the BFRPs at the early stage of immersion; the degradation reaction decreases with the increase in immersion time and the loss rate of flexural strength is initially higher, which is consistent with the findings of many studies [45].

**Figure 19:**
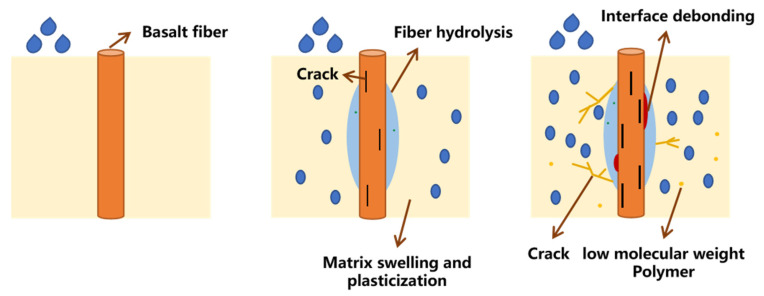
Moisture absorption mechanism of BFRPs.

Figure 19 shows the moisture absorption mechanism of BFRPs. In composite laminates, it is mainly the resin matrix and the interface that absorb water. The matrix swells and plasticizes after absorbing water, but the mismatch of moisture expansion between the fibers and the resin matrix causes the interface to debond, while the resin matrix starts to degrade and microcracks appear [46]. Studies have shown that basalt fiber has good water resistance, but its reaction with corrosive media in acidic and alkaline environments makes a large number of cracks appear on the fiber surface, which promotes the debonding of the interface [47].

**Figure 20:**
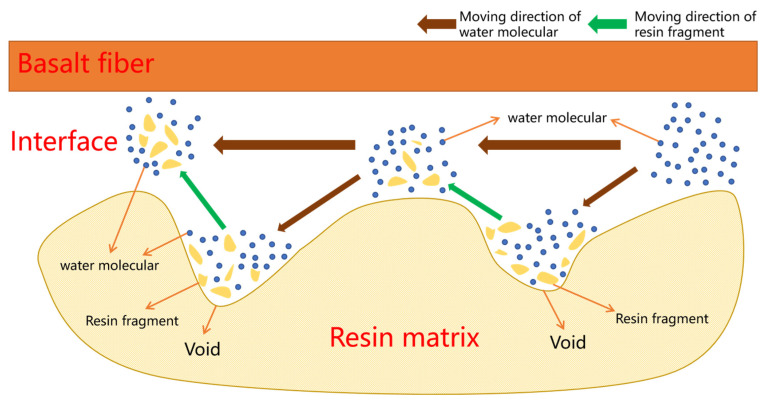
Interface situation of BFRPs.

Figure 20 shows the interface of the BFRPs. During the manufacturing process of the composite laminates, air bubbles are inevitably generated. None of the air bubbles can be removed during the solidification process. The solidification process also gives off a lot of heat inside the composite laminates. Heat dissipation further leads to geometric defects such as cracks and voids [48,49]. The free water molecules enter the defects in the resin matrix and form stress concentrations, making the micro-cracks and voids in the matrix spread out. At the same time, the appearance of cracks and voids makes water molecules spread in the matrix, and the gathering of water molecules at the cracks and voids will increase the swelling of the nearby matrix, which leads to greater internal stress, resulting in the debonding of the interface [50].

The three silane coupling agents improve the flexural properties of the BFRPs in the order of KH550, KH560, and A171, which is due to the differences in the mechanical properties of the BFRPs caused by the different organic groups in the side chains of the three silane coupling agents. KH550 contains amino groups, which are highly polar and have high surface energy and can react with the hydroxyl groups of the vinyl ester resin and form covalent bonds with each other. The unsaturated carbon–carbon double bond in A171 can react with the unsaturated double bond of vinyl ester resin and connect, but the overall modification effect is not as good as KH550 and KH560 [51,52]. The modification with silane coupling agent not only increases the flexural strength of the BFRPs but also slows down the degradation of its flexural properties in a corrosive aging environment, which can also be attributed to the increased interfacial bonding of the fibers to the resin and the tighter adhesion of the fibers to the resin, helping to reduce the volume occupied by cracks and voids formed during the fabrication and solidification of the composite [53]. Meanwhile, the hydroxyl group on the fiber surface reacts with the silicone, reducing its hydrophilicity and helping to slow down the debonding of the interface in corrosive aging environments [54].

**Figure 21:**
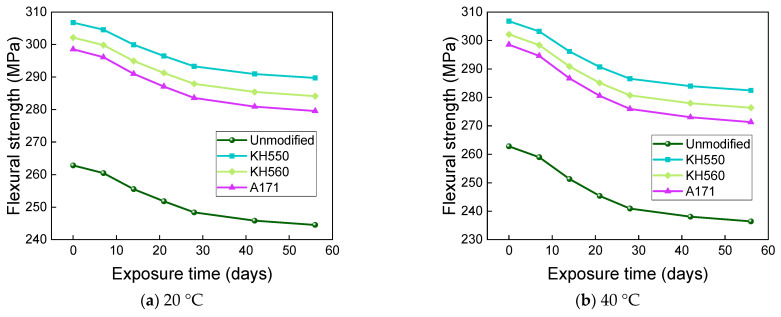

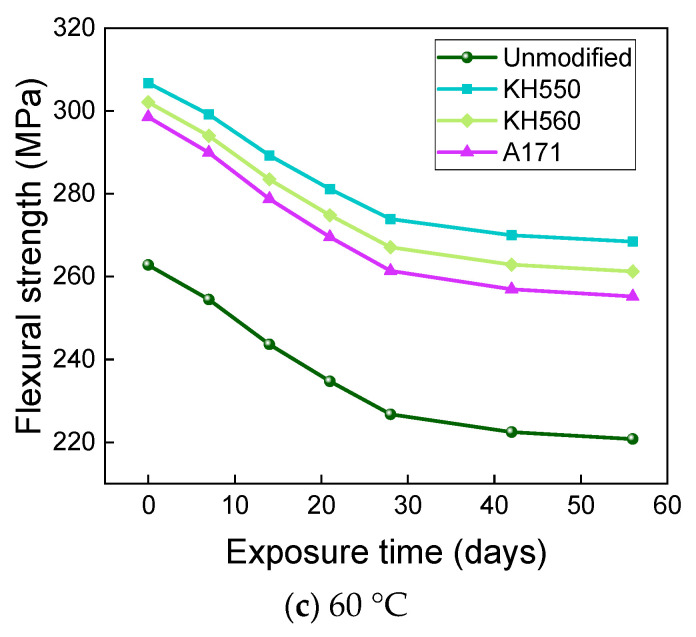
Flexural strength in a 3.5% NaCl seawater environment.

**Figure 22:**
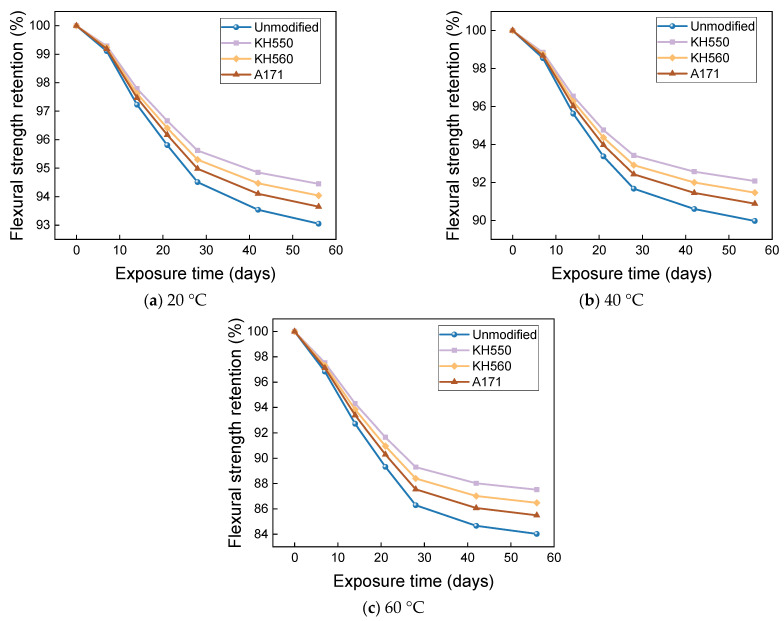
Flexural strength retention in a 3.5% NaCl seawater environment.

**Figure 23:**
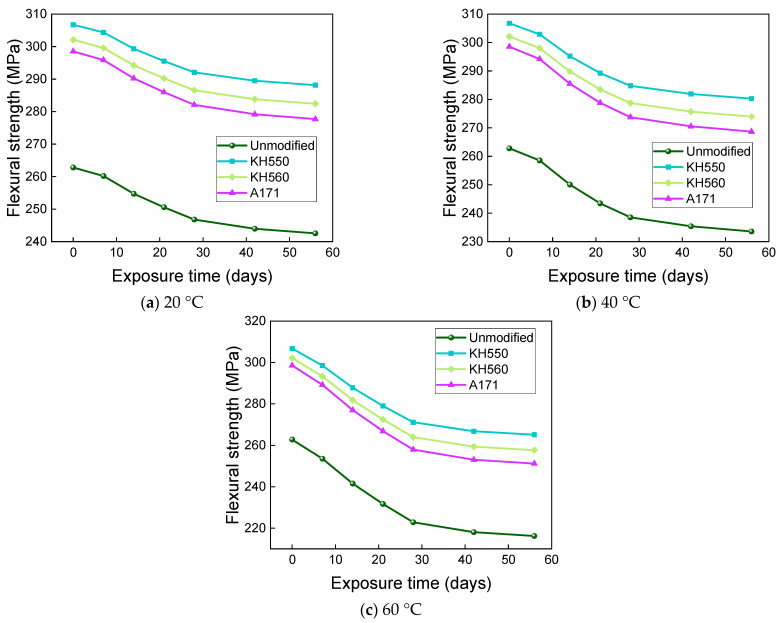
Flexural strength in a 10% NaCl high-concentration seawater environment.

**Figure 24:**
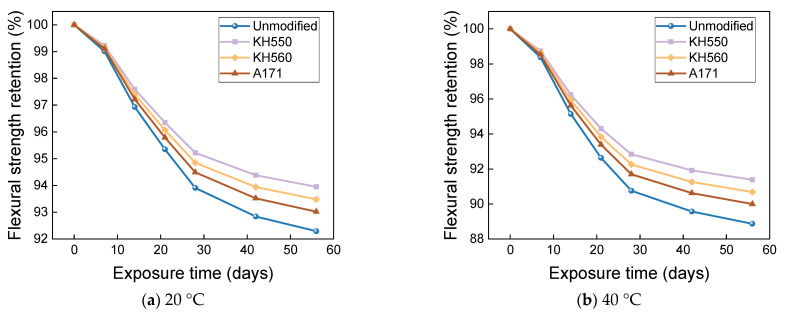

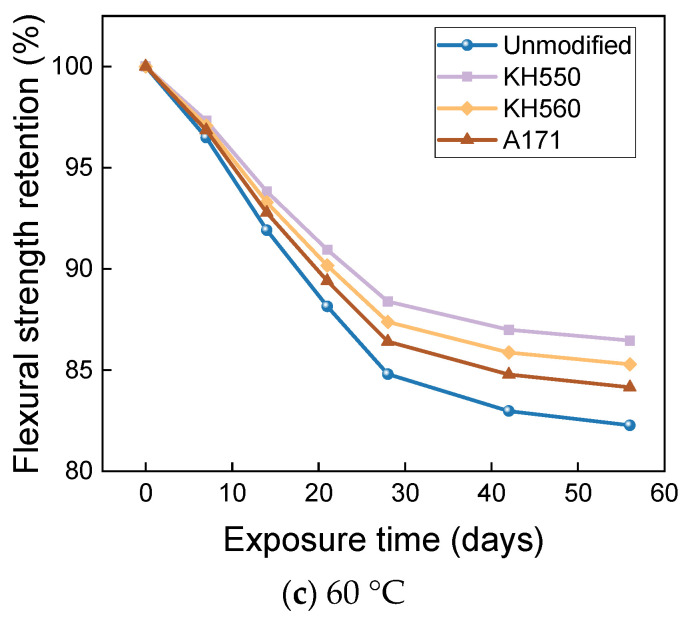
Flexural strength retention in a 10% NaCl high-concentration seawater environment.

The effects of different temperatures of 3.5% NaCl seawater environments on the flexural strength and flexural strength retention of the samples are shown in Figure 21 and Figure 22. After 56 days of immersion in seawater environments at 20 °C, the flexural strengths of the unmodified BFRPs and KH550, KH560, and A171 silane-coupling-agent- modified composites were 244.54 MPa, 289.71 Mpa, 284.12 Mpa, and 279.56 Mpa, which decreased by 6.95%, 5.55%, 5.96%, and 6.35% compared with the flexural strengths before immersion. The flexural strengths of the unmodified BFRPs and those modified with KH550, KH560, and A171 silane coupling agents were 236.44 MPa, 282.42 MPa, 276.33 MPa, and 271.31 MPa after 56 days of immersion in seawater environments at 40 °C, which were 10.03%, 7.93%, 8.54%, and 9.12% lower than the flexural strengths before immersion; after 56 days of immersion in seawater environments at 60 °C, the flexural strengths of the unmodified and KH550, KH560, and A171 silane-coupling-agent-modified BFRPs were 220.81 MPa, 268.44 MPa, 261.27 MPa, and 255.21 MPa, which decreased by 15.98%, 12.48%, 13.52%, and 14.51%. The effects of different temperatures of 10% NaCl high-concentration seawater environments on the flexural strengths and flexural strength retentions of the samples are shown in Figure 23 and Figure 24. After 56 days of immersion in high-concentration seawater environments at 20 °C, the flexural strengths of the unmodified and KH550, KH560, and A171 silane-coupling-agent-modified BFRPs were 242.55 MPa, 288.16 MPa, 282.41 MPa, and 277.70 MPa, which decreased by 7.71%, 6.05%, 6.52%, and 6.98% compared to the flexural strengths before immersion The flexural strengths of the unmodified BFRPs and those modified with KH550, KH560, and A171 silane coupling agents were 233.57 MPa, 280.28 MPa, 273.95 MPa, and 268.67 MPa after 56 days of immersion in high-concentration seawater environments at 40 °C, which were 11.13%, 8.62%, 9.32%, and 10.00% lower than the flexural strengths before immersion; after 56 days of immersion in high-concentration seawater environments at 60 °C, the flexural strengths of the unmodified and KH550, KH560, and A171 silane-coupling-agent-modified BFRPs were 216.22 MPa, 265.19 MPa, 257.67 MPa, and 251.22 MPa, which decreased by 17.73%, 13.54%, 14.71%, and 15.85%. The flexural strength of the samples in seawater environments gradually decreases with time, which is due to the presence of NaCl in the dissolved state in the form of cations and anions, since ions will penetrate the composite material together with water molecules. A large number of corrosive ions and water molecules gather on the surface of the fiber and matrix, which will generate pressure, constantly acting on the surface of the fiber and matrix and causing damage to the fiber, resin matrix, and its interface [55]. It can be found that the flexural strength retention of the samples in seawater environments does not change much compared to that in water environments, which indicates that BFRPs have relatively good corrosion resistance to seawater environments.

**Figure 25:**
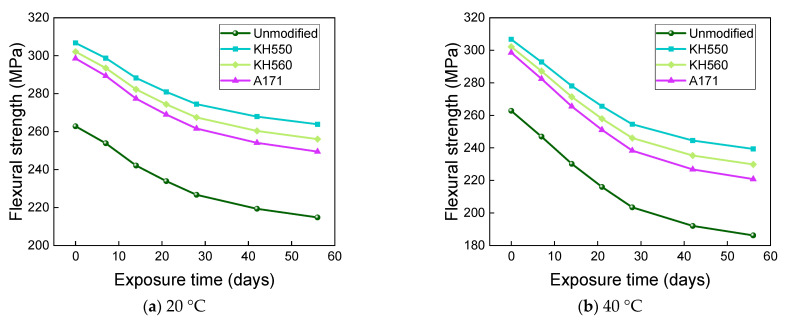

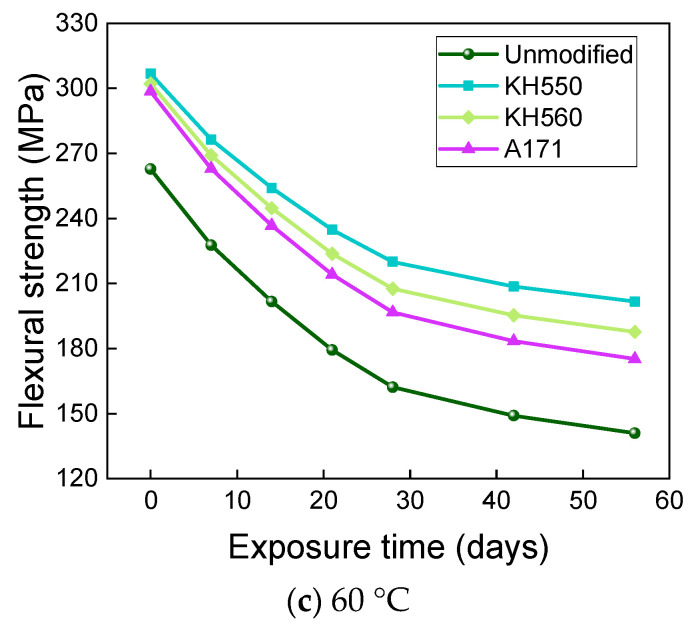
Flexural strength in a 10% H_2_SO_4_ acidic environment.

**Figure 26:**
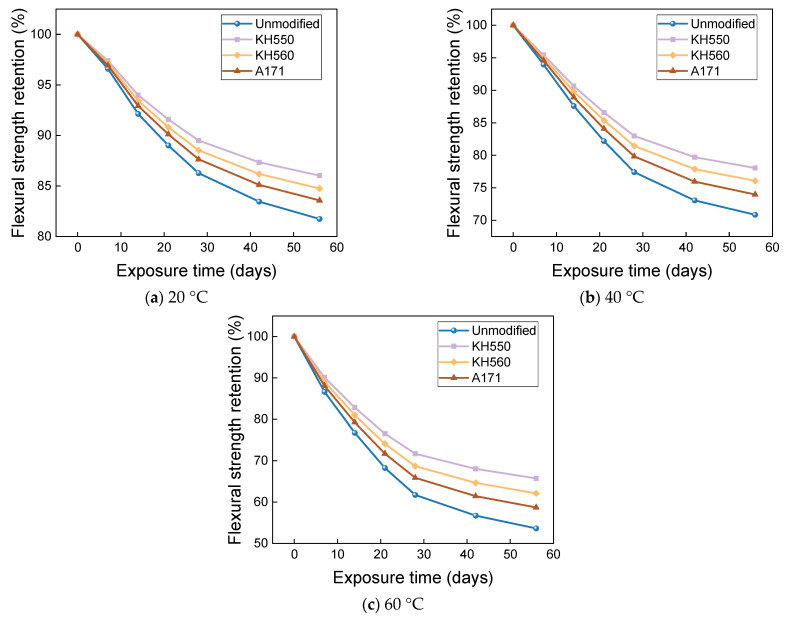
Flexural strength retention in a 10% H_2_SO_4_ acidic environment.

Figure 25 and Figure 26 show that the flexural strength and flexural strength retention of samples immersed in a 10% H_2_SO_4_ acid environment at different temperatures decreased significantly with time. The flexural strengths of the unmodified and KH550, KH560, and A171 silane-coupling-agent-modified BFRPs were 214.82 MPa, 263.88 MPa, 256.06 MPa, and 249.47 MPa after 56 days of immersion in the acid environments at 20 °C, which decreased by 18.26%, 13.97%, 15.25%, and 16.43% compared to the bending strength before immersion. The flexural strengths of unmodified and KH550, KH560, and A171 silane-coupling-agent-modified BFRPs were 186.20 MPa, 239.40 MPa, 229.82 MPa, and 220.82 MPa after 56 days of immersion in acidic environments at 40 °C, which decreased by 29.15%, 21.95%, 23.93%, and 26.03% compared to the flexural strength before immersion. The flexural strengths of the unmodified and KH550, KH560, and A171 silane-coupling-agent-modified BFRPs were 141.02 MPa, 201.69 MPa, 187.74 MPa, and 175.27 MPa after 56 days of immersion in acidic environments at 60 °C, which decreased by 46.34%, 34.25%, 37.86%, and 41.29%. The flexural strength retention of the samples in the acidic environment was lower than in the other corrosive aging environments but slightly higher than in the alkaline environment. This is because basalt fiber and vinyl ester resin are hydrolyzed under acidic conditions, by the resin hydrolysis formula as shown in Equation (1) [56], and resin ester group hydrolysis under acidic conditions is a reversible reaction, so the reaction is not complete. The SiO_2_ mesh structure of basalt fiber is more stable to acidic reagents, and the acid resistance of basalt fiber is stronger than the alkali resistance [57]. This results in higher retention of the flexural strength of the composite under acidic immersion than in alkaline environments.

Hydrolysis of resin matrix under acidic conditions.

(1) $${\mathrm{ROOR}}^{*}+H_{2}O\overset{H^{+}}{↔}\mathrm{RCOOH}+R^{*}\mathrm{OH}$$

**Figure 27:**
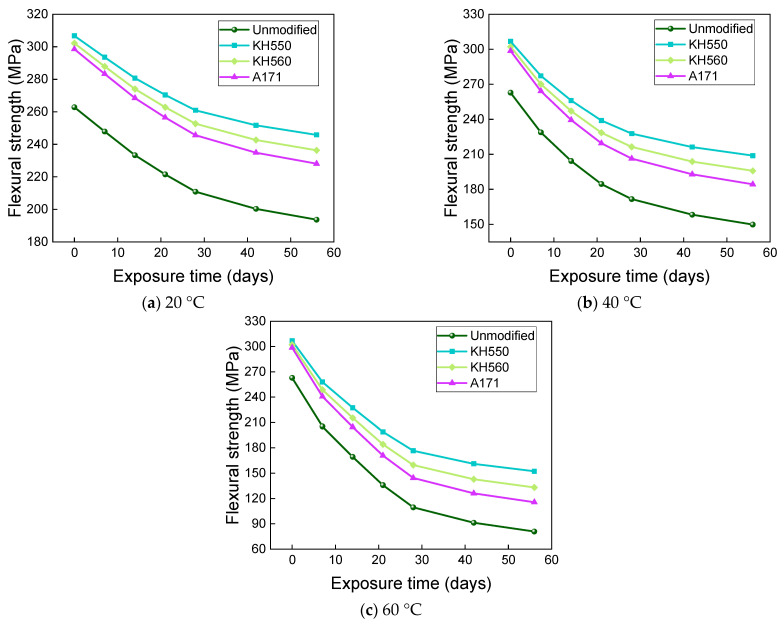
Flexural strength in a 10% NaOH alkaline environment.

**Figure 28:**
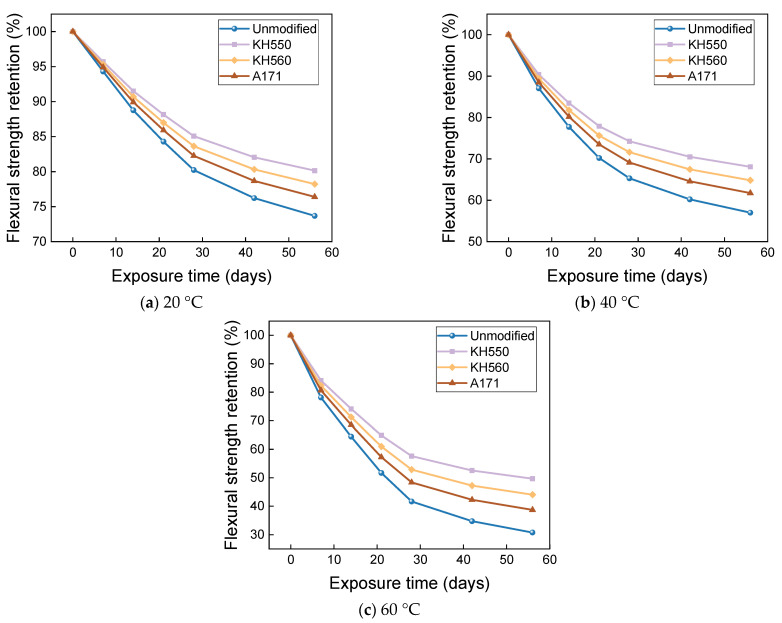
Flexural strength retention in a 10%NaOH alkaline environment.

Figure 27 and Figure 28 show that the flexural strength and bending strength retention of BFRPs showed a significant decrease with time under different temperatures of 10% NaOH alkaline environment immersion. The flexural strengths of the unmodified and KH550, KH560, and A171 silane-coupling-agent-modified BFRPs were 193.66 MPa, 245.80 MPa, 236.30 MPa, and 228.08 MPa after 56 days of soaking in alkaline environments at 20 °C, which decreased by 26.31%, 19.86%, 21.78%, and 23.60% compared with those before soaking. The flexural strengths of unmodified and KH550, KH560, and A171 silane-coupling-agent-modified BFRPs were 149.83 MPa, 208.89 MPa, 195.88 MPa, and 184.31 MPa after 56 days of immersion in alkaline environments at 40 °C, which decreased by 42.99%, 31.90%, 35.17%, and 38.26% compared to the flexural strength before immersion. After 56 days of soaking in alkaline environments at 60 °C, the flexural strengths of the unmodified and KH550, KH560, and A171 silane-coupling-agent-modified BFRPs were 80.84 MPa, 152.33 MPa, 133.10 MPa, and 115.60 MPa, which decreased by 69.24%, 50.34%, 55.95%, and 61.28%. The flexural strength retention of the BFRPs in alkaline environments is lower than in other corrosive aging environments. This is because the alkaline corrosive medium penetrates the interface by hygroscopic diffusion and reacts with the metal oxides of the basalt fibers, and the alkali solution destroys the Si-O-Si structure of the basalt fiber, as shown in Equation (2) [56], which causes significant damage to the fiber and accelerates the debonding of the interface [58]. On the other hand, the vinyl ester resin undergoes a hydrolysis reaction that damages the resin matrix, and the reaction is completely irreversible, as shown in Equation (3) [56]. These result in the lowest flexural strength retentions of the BFRPs in alkaline environments.

Hydrolysis of resin under alkaline environments.

(2) $${\mathrm{ROOR}}^{*}+\mathrm{NaOH}\to \mathrm{RCOONa}+R^{*}\mathrm{OH}$$

Reaction of Si-O-Si structure with alkaline environments.

(3) $$\mathrm{Si}-O-\mathrm{Si}+{\mathrm{OH}}^{-}\to \mathrm{SiOH}+{\mathrm{SiO}}^{-}$$

## 4. Conclusions

In this paper, the changes in the flexural properties of different types of silane coupling agent KH550-, KH560-, and A171-modified basalt-fiber-reinforced composites immersed in water at 20 °C, 40 °C, and 60 °C and 3.5% NaCl seawater, 10% NaCl high-concentration seawater, 10% H_2_SO_4_ acidic, and 10% NaOH alkaline environments were studied, and the following conclusions were drawn:

(a) The modification by silane coupling agents KH550, KH560, and A171 improved the interfacial bonding effect of BFRPs. Without the modification by silane coupling agents, there were large gaps between the fibers and the matrix, and the interfacial bonding effect was poor.
(b) The appearance of BFRPs varied greatly in different soaking environments. The samples soaked in water, 3.5% NaCl seawater, and 10% NaCl seawater environments did not show large changes, while the appearances of the samples soaked in acidic and alkaline environments showed great changes. Meanwhile, the appearance of BFRPs was greatly affected by the ambient temperature and soaking time, and a large number of white spots appeared on the surface of the samples after 56 days of soaking in the acidic and alkaline environments at 60 °C.
(c) The mass change of BFRPs accumulates gradually with soaking time, but the mass change rate decreases gradually with time. Temperature substantially enhances the mass change of BFRPs. The mass change of BFRPs in water at 20 °C, 40 °C, and 60 °C and 3.5% NaCl seawater, 10% NaCl seawater, and an alkaline environment mainly increased the mass. BFRPs exhibited mass gain in the acid environment at 20 °C, but a mass loss in the acid environments at 40 °C and 60 °C. The silane coupling agent modification reduced the mass change rate of BFRPs by forming a strong chemical bond connecting the fiber–resin matrix and enhancing the interfacial adhesion.
(d) The rate of the decrease in the flexural strength of BFRPs was positively correlated with the rate of mass change. The flexural strength gradually decreased with the increase in submergence time, but the decrease rate gradually slowed down with the increase in time. When the temperature increased, the flexural strength of the BFRPs decreased significantly. The alkaline environments had the greatest effect on the flexural properties of BFRPs compared to the water, seawater, and acid environments. The silane coupling agent modification improved the flexural properties of BFRPs by enhancing the interfacial bonding properties of the BFRPs and reduced the degradation of the flexural properties of BFRPs in corrosive aging environments. Considering the experimental results, the three silane coupling agents modified the corrosive aging performance of the composites in the order of KH550 > KH560 > A171.

## Figures and Tables

**Table 1:** Immersion conditions for five groups.

Group.	Immersion Environment	Temperature (°C)	Duration (Days)
1	Water	20, 40, 60	7, 14, 21, 28, 42, 56
2	3.5%NaCI	20, 40, 60	7, 14, 21, 28, 42, 56
3	10%NaCI	20, 40, 60	7, 14, 21, 28, 42, 56
4	10%H_2_SO_4_	20, 40, 60	7, 14, 21, 28, 42, 56
5	10%NaOH	20, 40, 60	7, 14, 21, 28, 42, 56

## Data Availability

Not applicable.
